# Characteristics of fracture in patients who firstly starts kidney replacement therapy in Korea: a retrospective population-based study

**DOI:** 10.1038/s41598-022-07178-4

**Published:** 2022-02-24

**Authors:** Youngrong Kim, Eunyoung Lee, Min-Jeong Lee, Bumhee Park, Inwhee Park

**Affiliations:** 1 Department of Nephrology, Ajou University School of Medicine, 164 Worldcup-ro, Yeongtong-gu, Suwon, Gyeonggi-do 16499 Republic of Korea; 2 Department of Biomedical Informatics, Ajou University School of Medicine, 164 Worldcup-ro, Yeongtong-gu, Suwon, Gyeonggi-do 16499 Republic of Korea; 3 Department of Medical Sciences, Biomedical Informatics, Graduate School of Ajou University, 164 Worldcup-ro, Yeongtong-gu, Suwon, Gyeonggi-do 16499 Republic of Korea; 4 Office of Biostatistics, Medical Research Collaborating Center, Ajou Research Institute for Innovative Medicine, Ajou University Medical Center, 164 Worldcup-ro, Yeongtong-gu, Suwon, Gyeonggi-do 16499 Republic of Korea

**Keywords:** Diseases, Nephrology, Risk factors

## Abstract

The incidence of fractures in patients with end-stage kidney disease (ESKD) is high which is associated with high morbidity and mortality. Since fractures are preventable diseases to some extent, epidemiologic studies are needed a lot. The aim of this study is to explore the epidemiology of fractures by modality of kidney replacement therapy (KRT). We performed a retrospective analysis of 52,777 patients dependent on KRT from 2008 to 2017 using the National Health Insurance System of Republic Korea. Fractures were occurred in 8995 (17.04%) of 52,777 patients with ESKD. Hemodialysis and kidney transplant patients had the highest (57.4 per 1000 person-year) and the lowest (25.2 per 1000 person-year) incidence rate, respectively. The two most common fracture sites were the lower limb and upper limb, regardless of KRT modality. The first fractures were about 2.55 ± 2.07 years after KRT initiation, the earliest in Hemodialysis patients. Diabetes mellitus, cerebrovascular disease, chronic lung and liver disease were risk factors of fractures. The use of steroids, anti-osteoporosis medications, and some classes of psychotropics and opioids was associated with an elevated risk. The results of this study inform the understanding of fractures in KRT patients.

## Introduction

The risk of fracture in patients undergoing kidney replacement therapy (KRT) is known to be higher than in the general population^1^. The fractures in KRT patients result in high mortality and low quality of life, and they impose a severe economic burden. In this regard, most of the existing studies are limited to hip fracture or report in one of KRT modality. Based on recent meta-analysis of 14 published cohort studies evaluating the risk of hip fracture in patients undergoing dialysis, the pooled relative risk was reported to be 5.8 compared to the general population^2^. Lin et al.^3^ reported that patients on hemodialysis (HD) showed a 31% higher incidence of hip fracture than those on peritoneal dialysis (PD). Increasing age, female gender, prior hip fracture, osteoporosis, diabetes mellitus (DM) and liver cirrhosis were identified as additional risk factors for hip fractures in patients with end-stage kidney disease (ESKD)^3^. However, Stehman-Breen et al.^4^ reported that the mode of dialysis and DM were not important predictors of hip fractures. A population-based cohort study describing fracture risk in patients with chronic kidney disease (CKD) compared the fracture risk of patients with CKD before dialysis with patients undergoing dialysis, which revealed a higher risk (hazard ratio [HR] 1.16; 95% confidence interval [CI] 1.12 to 1,21; *P* < 0.001) in the latter group^5^. Fractures can be prevented by delineating their frequency and location in these patients as well as the medications and modalities of KRT used to some extent. To do so, we should understand the characteristics of fractures and establish a fracture prevention strategy. However, studies on the epidemiology of fractures are lacking in general, and the previous studies are mostly studies on one or two modalities of KRT or fractures at specific sites, so comprehensive studies are needed. Therefore, we analyzed the fracture characteristics including incidence rate (IR), the time to first fracture event, risk factors, concomitant medications, and locations of fracture by KRT modiality using the nation-wide Health Insurance Review and Assessment Service (HIRA) database of Republic Korea.

## Materials and methods

### Data source

All data were obtained from the HIRA database, which contains the epidemiologic and clinical data from the National Health Insurance System (NHIS). The NHIS covers more than 98% of the Korean population^6^. This study was conducted in accordance with the Declaration of Helsinki. The HIRA Database was fully anonymized and the requirement of informed consent was waived by the ethics committee of the Institutional Review Board of Ajou University School of Medicine (IRB No. AJIRB-MED-EXP-18–487).

### Study population

**Figure 1:**
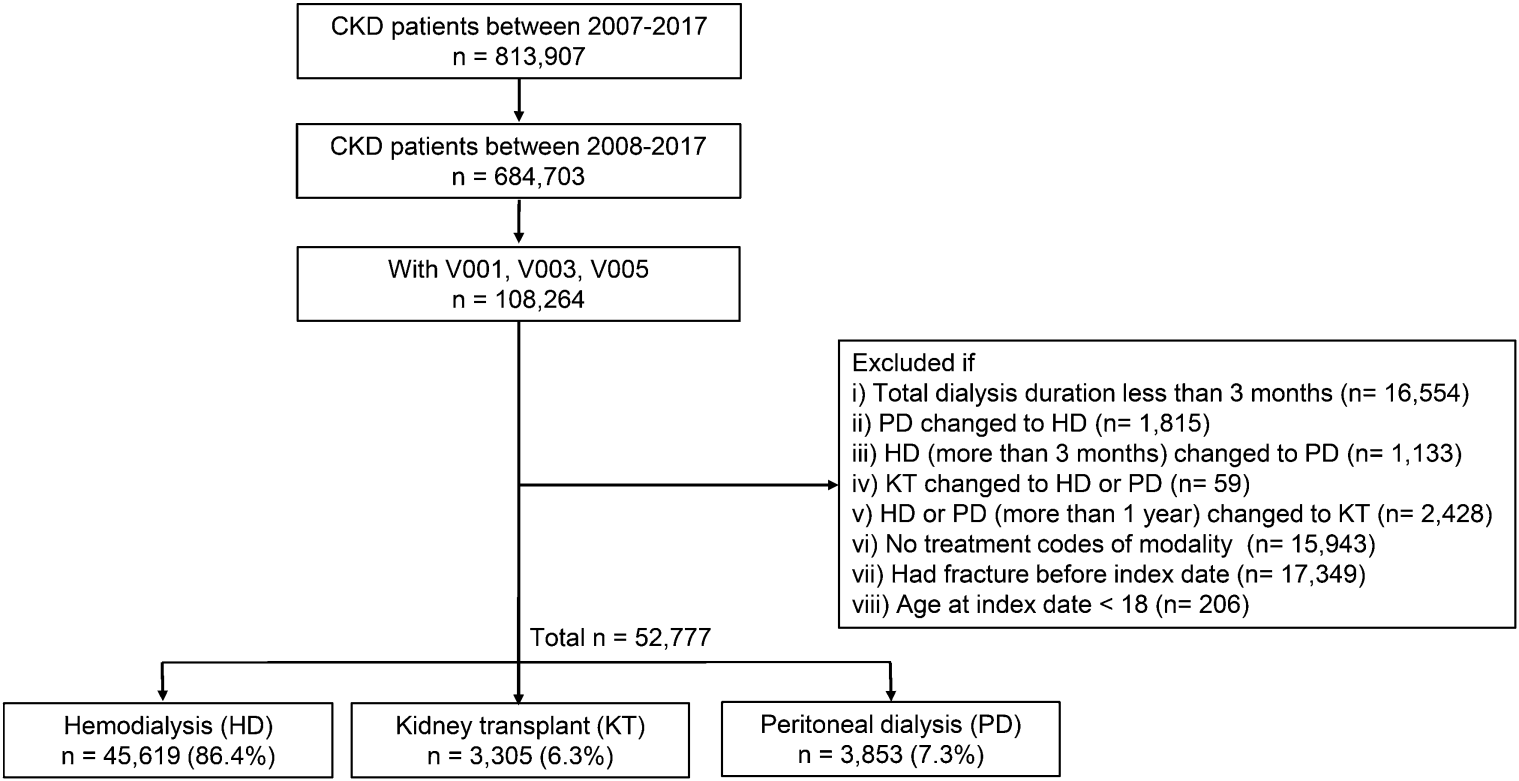
Flow chart outline of the study design. *CKD* chronic kidney disease.

We initially collected patients’ data using the diagnostic code of CKD (International Code of Diagnosis, 10th edition, ICD-10, N18.1–N18.5, and N18.9) from 2007 to 2017 from the HIRA database. We included only patients with newly diagnosed ESKD during this study period (2008–2017) by excluding patients carrying the diagnostic codes in 2007. Among them, patients with specific codes of V001 (hemodialysis), V003 (peritoneal dialysis) and V005 (kidney transplant) were extracted to establish the ESKD diagnosis and then classified into three groups based on the mode of KRT: hemodialysis (HD), peritoneal dialysis (PD), and kidney transplant (KT). Before classification, however, to ensure the accuracy of analysis, we excluded some of the patients based on the following criteria: (1) total duration of dialysis less than 3 months; (2) switch from PD to HD; (3) switch to PD after treatment with HD for more than three months; (4) change from KT to HD or PD due to graft failure; and (5) undergoing transplantation more than one year after the first dialysis (for KT patients). The date of dialysis initiation or kidney transplant was considered as an index date. Patients without treatment codes of modality, or aged under 18 years at index date, or patients who had a fracture before the index date were also excluded (Fig. 1).

### Ascertainment of fractures

We used ICD-10 codes for identifying fracture events and defining specific sites of fracture including hip, pelvis, upper limb, lower limb, vertebra, rib/sternum, and skull. Multiple anatomically distant sites of fracture in one patient were recorded as separate fracture events.

### Ascertainment of risk factors

Covariates identified as potential risk factors for fracture included age, sex, modality of KRT, comorbidities (diabetes, hypertension, cardiovascular disease, cerebrovascular disease, chronic lung and liver disease), and medications (steroids, Vitamin D and analogs, phosphate binders, anti-osteoporotic agents, anti-depressants, opioids, and gabapentinoids). Patients were considered to manifest comorbidities if they had diagnostic codes more than twice within one year before the index date. Baseline medications were defined by at least 30 days of prescriptions filled within one year before the index date.

### Statistical analysis

The patients' baseline demographics were compared based on KRT modalities and their differences were tested by analysis of variance (ANOVA) or chi-squared test. Chi-squared test was used to compare the frequency of fracture incidence at specific fracture sites among the different KRT groups. The incidence rates (IR) of fracture were calculated as the total number of fractures divided by the sum of all follow-up person-years (PY), adjusted by age and sex, and expressed per 1000 PY with 95% CIs. The cumulative incidence plot was used to assess the difference in fracture risk between the three groups. Cox proportional-hazards regression models were used to estimate the risk factors for fractures. The proportional hazard assumptions were tested and visualized by Shoenfeld residuals. Covariates used in univariable and multivariable analysis included age at index, sex, KRT modality, comorbidities, and medications that patients took at baseline. The HR of fractures and their 95% CIs were estimated for each covariate. All statistical analyses were based on two-sided inferences and were performed using SAS statistical software, version 9.4 (SAS Institute, Cary, NC, USA). Only complete cases were used in the analysis. *P* values under 0.05 were regarded as statistically significant.

### Ethical standards

This study was conducted in accordance with the Declaration of Helsinki and approved by the Institutional Review Board of Ajou University College of Medicine (IRB No. AJIRB-MED-EXP-18–487).

### Informed consent

The HIRA Database was fully anonymized. The requirement of informed consent was waived.

## Results

### Demographic characteristics of the study population

We identified 52,777 patients on KRT at the initiation of the study including 45,619 on HD, 3853 on PD, and 3305 on KT. The demographic characteristics of patients at initiation are listed in Table 1. The use of KRT among males was higher than in females with ESKD. The overall follow-up period (mean ± standard deviation [SD]) was 4.50 ± 2.49 years. Mean age (SD) at the index date was 59.21 (14.19) years. The HD group contained the oldest patients, aged 60.78 (13.69) years. The KT group was the youngest, with patients’ age 44.62 (11.62) years. The average time from the index date to fracture diagnosis was 2.55 years overall, and all KRT groups showed an average time ranging from 2.5 to 3 years. In terms of comorbidities, patients undergoing HD showed highest prevalence of DM (65.9%), cardiovascular disease (25.2%), cerebrovascular disease (16.2%), chronic lung disease (21.7%) and chronic liver disease (4.4%). The use of corticosteroids (37.2%), vitamin D and its analogs (30.2%), and phosphate binders (74.1%) was the highest in the KT group, whereas the use of anti-osteoporotic medications (1.5%), anti-depressants (9.4%), opioids (2.2%) and gabapentinoids (3.7%) was the lowest.

**Table 1:** Demographic characteristics of the study population stratified by kidney replacement therapy.

Characteristics	Overall (n = 52,777)	HD (n = 45,619)	KT (n = 3305)	PD (n = 3853)
Observation duration, mean (SD), years	4.50 (2.49)	4.48 (2.52)	4.51 (2.34)	4.66 (2.35)
**Age at index date, mean (SD), years**	59.21 (14.19)	60.78 (13.69)	44.62 (11.62)	53.16 (13.25)
18–29, n (%)	1335 (2.53)	794 (1.74)	385 (11.65)	156 (4.05)
30–39, n (%)	3695 (7.00)	2526 (5.54)	738 (22.33)	431 (11.19)
40–49, n (%)	8191 (15.52)	6311 (13.83)	943 (28.53)	937 (24.32)
50–59, n (%)	12,609 (23.89)	10,595 (23.22)	930 (28.14)	1084 (28.13)
60–69, n (%)	12,625 (23.92)	11,564 (25.35)	289 (8.74)	772 (20.04)
≥ 70, n (%)	14,322 (27.14)	13,829 (30.31)	20 (0.61)	473 (12.28)
Age at fracture diagnosis, mean (SD), years^b^	63.58 (12.98)	64.42 (12.66)	49.30 (11.56)	58.07 (12.96)
Time from index date to fracture diagnosis, mean (SD), years^b^	2.55 (2.07)	2.52 (2.07)	2.85 (1.90)	2.78 (2.14)
Sex, Male, n (%)	33,046 (62.61)	28,918 (63.39)	1909 (57.76)	2219 (57.59)
**Comorbidities**^c^				
Diabetes, n (%) E10–E14	33,860 (64.16)	30,061 (65.90)	1501 (45.42)	2298 (59.64)
Hypertension, n (%) I10–I15	47,863 (90.69)	41,189 (90.29)	3141 (95.04)	3533 (91.69)
Cardiovascular, n (%) I20–I25	12,977 (24.59)	11,500 (25.21)	536 (16.22)	941 (24.42)
Cerebrovascular, n (%) I60–I69	7950 (15.06)	7385 (16.19)	191 (5.78)	374 (9.71)
Chronic lung disease, n (%) J40–J47	10,986 (20.82)	9905 (21.71)	494 (14.95)	587 (15.23)
Chronic liver disease, n (%) K70.3, K70.4, K72.1, K73, K74	2239 (4.24)	2022 (4.43)	69 (2.09)	148 (3.84)
**Medications at baseline**				
Steroid	5634 (10.68)	4099 (8.99)	1228 (37.16)	307 (7.97)
Vitamin D and its analog	7848 (14.87)	5733 (12.57)	999 (30.23)	1116 (28.96)
Phosphate binder	23,031 (43.64)	18,046 (39.56)	2449 (74.10)	2536 (65.82)
Anti-osteoporotic agent	1208 (2.29)	1095 (2.40)	48 (1.45)	65 (1.69)
Anti-depressant	7552 (14.31)	6692 (14.67)	309 (9.35)	551 (14.3)
Opioids	2812 (5.33)	2621 (5.75)	73 (2.21)	118 (3.06)
Gabapentinoids	4517 (8.56)	4149 (9.09)	123 (3.72)	245 (6.36)

^a^P value was based on analysis of variance (ANOVA), otherwise chi-square test.

^b^Analyzed in patients with fracture (n = 8995) only.

^c^Comorbidities and medications were observed 1 year before index date; dialysis date or kidney transplant date.

*HD* hemodialysis, *KT* kidney transplant, *PD* peritoneal dialysis, *SD* standard deviation.

### Frequency and IR of fractures by site and modality in patients with ESKD

**Figure 2:**
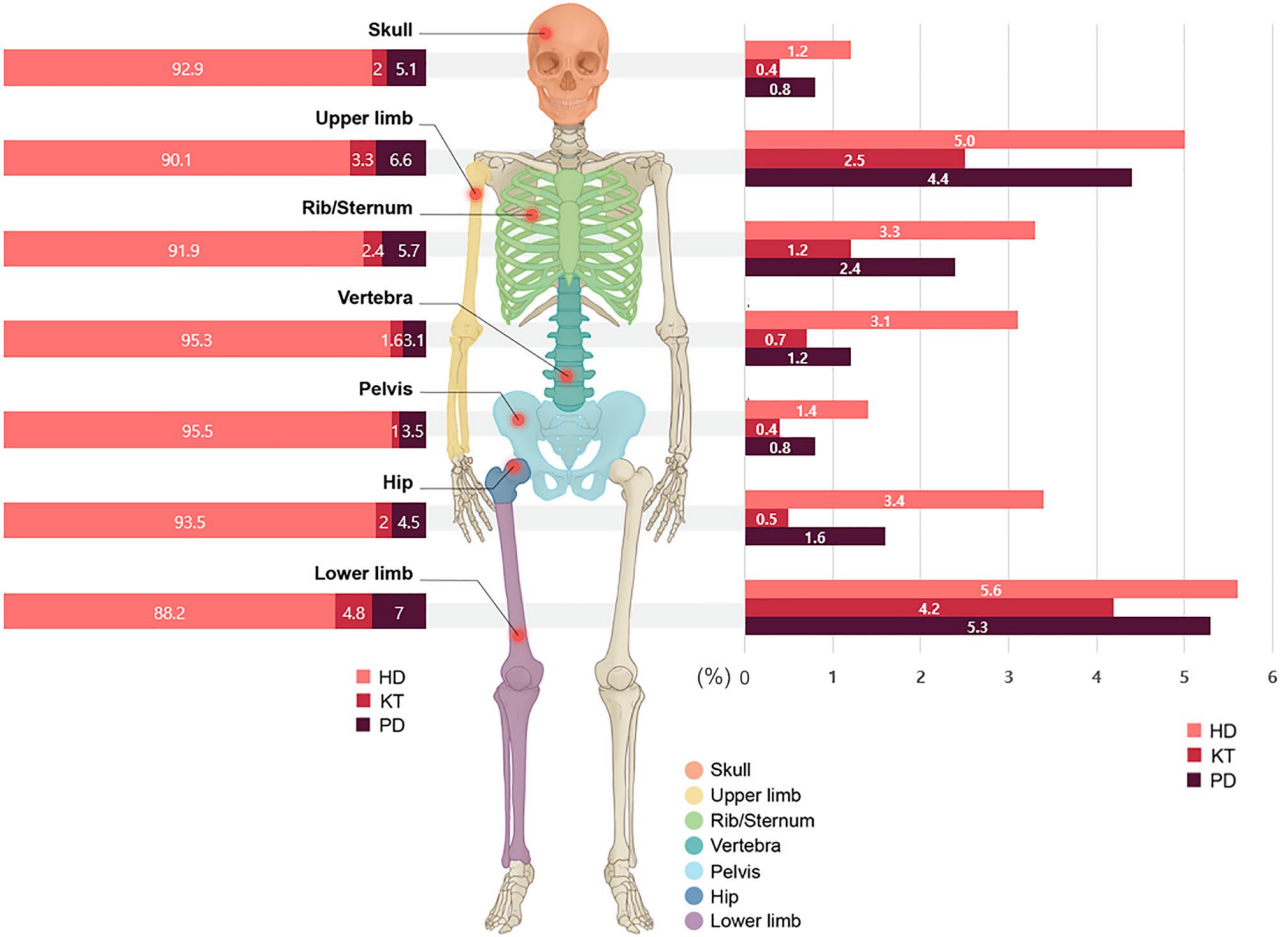
Proportion (%) of specific kidney replacement therapy modality used at each fracture site (left panel) and the frequency (%) of fracture based on anatomical site according to kidney replacement therapy (right panel). *HD* hemodialysis, *KT* kidney transplant, *PD* peritoneal dialysis.

A total of 8995 fracture events were identified during the study period with an overall IR of 53.7 per 1000 PY. The overall fracture IR were 57.4 per 1000 PY, 25.2 per 1000 PY and 38.5 per 1000 PY for the HD, KT, and PD groups, respectively. The frequency of overall fractures and each fracture site are listed in Table 2 and Fig. 2 (right panel), based on the KRT modality used. The two most common fracture sites were the lower limb and upper limb, followed by rib/sternum, hip and vertebrae. However, these last three fracture sites differ to a greater or lesser degree, depending on KRT modality. Accordingly, the analysis of all the fracture IRs is presented in Table 3. The HD group had the highest IR, not only for overall fractures but also for each site of fracture (Table 3, Fig. 2 [left panel]). Even after adjusting by age and sex, the result has not been changed.

**Table 2:** Frequency of radiological fractures in kidney replacement therapy populations during follow-up.

	Overall (n = 52,777)	HD (n = 45,619)	KT (n = 3305)	PD (n = 3853)	P value^a^
All fractures, n (%)	8995 (17.04)	8199 (17.97)	282 (8.53)	514 (13.34)	< 0.001
Hip, n (%)	1616 (3.06)	1540 (3.38)	16 (0.48)	60 (1.56)	< 0.001
Pelvis, n (%)	705 (1.34)	659 (1.44)	14 (0.42)	32 (0.83)	< 0.001
Upper limb, n (%)	2551 (4.83)	2299 (5.04)	83 (2.51)	169 (4.39)	< 0.001
Lower limb, n (%)	2897 (5.49)	2556 (5.6)	138 (4.18)	203 (5.27)	0.002
Vertebra, n (%)	1493 (2.83)	1423 (3.12)	24 (0.73)	46 (1.19)	< 0.001
Rib/Sternum, n (%)	1622 (3.07)	1491 (3.27)	39 (1.18)	92 (2.39)	< 0.001
Skull, n (%)	592 (1.12)	550 (1.21)	12 (0.36)	30 (0.78)	< 0.001

^a^P value was based on Chi-square test.

*HD* hemodialysis, *KT* kidney transplant, *PD* peritoneal dialysis.

**Table 3:** Time to first fracture identification and fracture site among end-stage kidney disease patients stratified by KRT.

Fracture sites	KRT modality	Event, n	Time to the identification of fracture, years, mean (SD)	PY	IR per 1000 (95% CI)	Age and sex adjusted IR per 1000 (95% CI)
All fractures		8995	2.55 (2.07)	167,349.2	53.7 (52.65, 54.87)	-
All sites	HD	8199	2.52 (2.07)	142,834	57.4 (56.17, 58.65)	47.1 (36.37, 61.05)
	KT	282	2.85 (1.90)	11,180	25.2 (22.41, 28.30)	27.8 (21.46, 36.14)
	PD	514	2.78 (2.14)	13,335	38.5 (35.32, 41.99)	37.4 (28.88, 48.51)
Hip	HD	1540	2.70 (2.18)	159,017	9.7 (9.21, 10.18)	4.5 (1.78, 11.56)
	KT	16	2.83 (2.26)	11,806	1.4 (0.80, 2.15)	0.6 (0.25, 1.5)
	PD	60	3.02 (2.20)	14,284	4.2 (3.23, 5.37)	1.6 (0.69, 3.73)
Pelvis	HD	659	2.90 (2.26)	160,258	4.1 (3.81, 4.44)	3.2 (1.37, 7.59)
	KT	14	3.54 (1.91)	11,812	1.2 (0.67, 1.94)	1.3 (0.51, 3.15)
	PD	32	2.55 (1.67)	14,315	2.2 (1.56, 3.12)	1.2 (0.49, 2.99)
Upper limb	HD	2299	2.82 (2.17)	156,138	14.7 (14.13, 15.34)	12.9 (10.13, 16.48)
	KT	83	2.98 (2.23)	11,643	7.1 (5.71, 8.79)	7.7 (6.02, 9.84)
	PD	169	2.65 (1.76)	13,983	12.1 (10.36, 14.02)	12.3 (9.65, 15.71)
Lower limb	HD	2556	2.94 (2.17)	155,246	16.5 (15.84, 17.11)	16.9 (12.02, 23.73)
	KT	138	2.84 (2.17)	11,504	12.0 (10.12, 14.13)	9.3 (6.58, 13.24)
	PD	203	2.94 (1.76)	13,947	14.6 (12.65, 16.66)	13.9 (9.91, 19.63)
Vertebra	HD	1423	2.53 (2.07)	158,792	9.0 (8.51, 9.44)	3.2 (1.34, 7.76)
	KT	24	2.97 (2.48)	11,795	2.0 (1.33, 2.98)	2.9 (1.1, 7.59)
	PD	46	2.80 (1.95)	14,284	3.2 (2.39, 4.26)	1.5 (0.61, 3.74)
Rib/sternum	HD	1491	2.77 (2.13)	158,365	9.4 (8.95, 9.90)	5.7 (2.75, 11.67)
	KT	39	3.34 (2.19)	11,773	3.3 (2.39, 4.48)	3.7 (1.62, 8.29)
	PD	92	3.43 (2.23)	14,185	6.5 (5.26, 7.92)	4.2 (1.96, 9.12)
Skull	HD	550	2.74 (2.21)	160,400	3.4 (3.15, 3.73)	3.3 (1.51, 7.27)
	KT	12	3.08 (1.99)	11,823.5	1.0 (0.55, 1.73)	0.7 (0.33, 1.73)
	PD	30	3.23 (1.45)	14,307.83	2.1 (1.44, 2.96)	2.3 (1.06, 5.06)

*CI* confidence interval, *HD* hemodialysis, *IR* incidence rate, *KRT* kidney replacement therapy, *KT* kidney transplant, *PD* peritoneal dialysis, *PY* person-years, *SD* standard deviation.

### Time to initial detection of fractures in ESKD populations

We also analyzed the time of the first fracture using the index date (Table 3). The mean time (mean ± SD) of the first fracture was 2.52 ± 2.07, 2.85 ± 1.90, and 2.78 ± 2.14 years for the HD, KT and PD groups, respectively, for all fracture sites. Most of the fractures occurred the earliest in the HD group.

### Cox proportional-hazards regression analysis of risk factors for fracture in patients with ESKD

**Figure 3:**
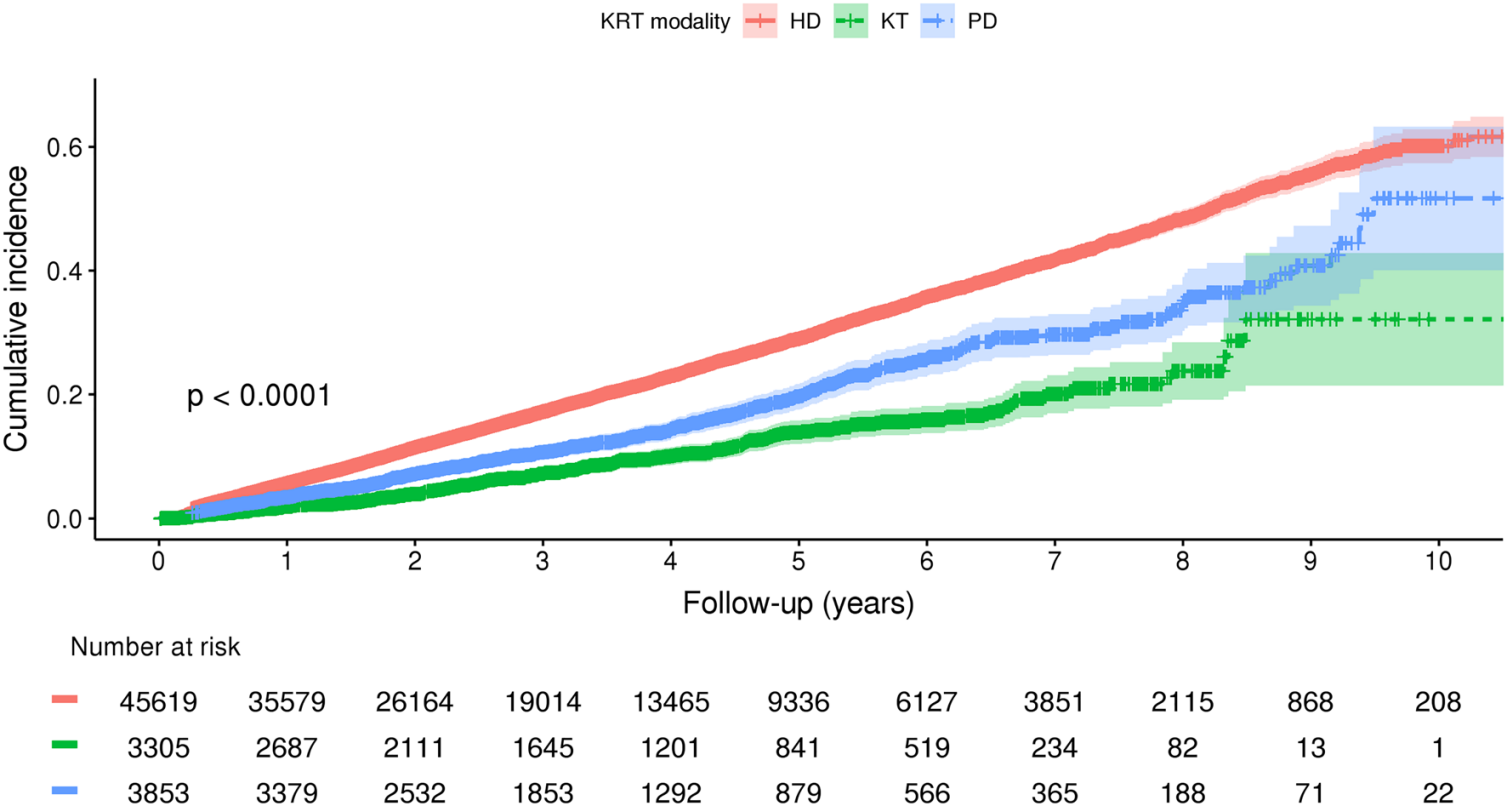
Cumulative incidence of fracture risk in patients with end-stage kidney disease undergoing KRT. *KRT* kidney replacement therapy, *HD* hemodialysis, *KT* kidney transplant, *PD* peritoneal dialysis.

As illustrated in Fig. 3, the highest cumulative incidence of fractures stratified by KRT modality was observed in HD, followed by PD and KT. The Cox proportional-hazards regression model was performed to investigate the risk factors for fractures (Table 4). All variables used in univariable analysis were included in the multivariable analysis following the stepwise selection methods. Based on this study, increasing age (HR, 1.02; 95% CIs, 1.02 to 1.03), female gender (HR, 1.46; 95% CIs, 1.40 to 1.52), undergoing HD (HR, 1.49 relative to KT; 95% CIs, 1.31 to 1.68) or PD (HR 1.22 relative to KT; 95% CIs, 1.06 to 1.42) are risk factors for fracture.

**Table 4:** HR and their 95% CI based on univariable and multivariable Cox proportional-hazards regression model.

	Univariable analysis				Multivariable analysis			
	HR	95% CI		*P* value	Adjusted HR	95% CI		*P* value
Age at index, years	1.03	1.03	1.03	< 0.001	1.02	1.02	1.03	< 0.001
Sex, female	1.42	1.36	1.48	< 0.001	1.46	1.40	1.52	< 0.001
**KRT modality**								
HD	2.27	2.02	2.56	< 0.001	1.49	1.31	1.68	< 0.001
PD	1.53	1.32	1.76	< 0.001	1.22	1.06	1.42	0.007
KT (Reference)	1.00				1.00			
**Comorbidities**								
Diabetes	1.58	1.51	1.65	< 0.001	1.39	1.33	1.47	< 0.001
Hypertension	1.25	1.16	1.34	< 0.001	0.90	0.83	0.97	0.006
Cardiovascular	1.27	1.21	1.33	< 0.001	1.01	0.96	1.06	0.690
Cerebrovascular	1.37	1.30	1.45	< 0.001	1.09	1.03	1.15	0.005
Chronic lung disease	1.44	1.37	1.51	< 0.001	1.18	1.12	1.24	< 0.001
Chronic liver disease	1.28	1.16	1.41	< 0.001	1.28	1.16	1.41	< 0.001
**Medication at baseline**								
Steroid	0.97	0.91	1.04	0.456	1.12	1.05	1.21	0.001
Vitamin D and its analog	0.83	0.78	0.89	< 0.001	0.92	0.86	0.98	0.016
Phosphate binders	0.84	0.81	0.88	< 0.001	0.96	0.92	1.00	0.040
Anti-osteoporotic medications	1.54	1.35	1.75	< 0.001	1.17	1.02	1.33	0.021
Anti-depressants	1.43	1.35	1.51	< 0.001	1.19	1.13	1.26	< 0.001
Opioids	1.58	1.45	1.73	< 0.001	1.18	1.08	1.30	< 0.001
Gabapentinoids	1.48	1.38	1.59	< 0.001	1.21	1.13	1.30	< 0.001

*CI* confidence interval, *HD* hemodialysis, *HR* hazard ratio, *KRT* kidney replacement therapy, *KT* kidney transplant, *PD* peritoneal dialysis.

In terms of comorbidities, DM (HR, 1.39; 95% CIs, 1.33 to 1.47), cerebrovascular disease (HR, 1.09; 95% CIs, 1.03 to 1.15), chronic lung disease (HR, 1.18; 95% CIs, 1.12 to 1.24) and chronic liver disease (HR, 1.28; 95% CIs, 1.16 to 1.41) were the other identified risk factors along with the use of steroids (HR, 1.12; 95% CIs, 1.05 to 1.21), anti-osteoporotic medications (HR, 1.17; 95% CIs, 1.02 to 1.33), anti-depressants (HR, 1.19; 95% CIs, 1.13 to 1.26), opioids (HR,1.18; 95% CIs, 1.08 to 1.30) and gabapentinoids (HR, 1.21; 95% CIs, 1.33 to 1.47). In contrast, treatment with vitamin D and vitamin D analogs (HR, 0.92; 95% CIs, 0.86 to 0.98) and phosphate binders (HR 0.96; 95% CIs, 0.92 to 1.00) was associated with a decreased risk of fracture.

## Discussion

This large-scaled nation-wide study demonstrated that HD was associated with the highest IR of fractures (57.4 per 1,000 PY), and the two most common fracture sites were the lower limb and upper limb, followed by rib/sternum, hip and vertebrae. The average time of first fractures was about 2.55 ± 2.07 years from the index date and fractures occurred the earliest in HD patients. The findings suggest that exposure to HD and a diagnosis of DM, cerebrovascular disease, chronic lung and liver disease were associated with increased risk of fractures. Additional risk factors included intake of medications like steroids, anti-osteoporotic drugs, anti-depressants, opioids and gabapentinoids.

The overall fracture IR of patients with ESKD in this study was 53.8 per 1000 PY, which was lower than in a previous study conducted in the West of Scotland^7^. Dey et al.^7^ reported that the overall fracture IR was 62.8 per 1000 PY in all ESKD patients, 99.2 per 1000 PY for those exposed to HD, 57.6 per 1,000 PY for those treated with PD, and 37.6 per 1000 PY for those undergoing KT. As for the hip fractures, however, our data showed an IR of 8.7 per 1000 PY for all patients with ESKD, 9.7 per 1000 PY for patients treated with HD, which was comparable to data reported in previous studies^2,8^. Our data are consistent with other reports, showing a higher fracture IR in ESKD patients than in the general population^1,2,9,10^. Based on the Korean population-based hip-fracture study by Choi et al.^11^ using the HIRA database, the age-adjusted IR of hip fractures was 2.1 per 1000 PY for men, and 3.1 per 1000 PY for women, both of which were far lower than the IR of hip fracture in ESKD patients, analyzed in this study.

This study was consistent with previous reports indicating that the HD group had the highest IR of fractures among all ESKD patients^2,3,7,12^. Thus, we reinforced the significance of KRT modality as a risk factor for fracture in the presence of other comorbidities and specific medications^4,7,8,10,12–15^. Based on our data, further attention is needed to prevent fractures in dialyzed patients, especially those on HD. The mean age at the index date was the highest in patients treated with HD. The highest prevalence of DM, cardiovascular disease, chronic lung and liver disease was identified in HD patients. Thus, since patients undergoing HD were mostly elderly suffering from inactivity, frailty, and multiple comorbidities, they were vulnerable to falls and fractures^8,14^. Hemodynamic instability and post-dialysis hypotension are also presumed to contribute to falls and fractures in HD populations. Additionally, metabolic abnormalities may further explain the increased IR of fractures in HD patients. These may include routine use of heparin, chronic acidosis, and a higher level of parathyroid hormone (PTH)^14^. Stein et al.^16^ reported that these factors decreased the bone mass and worsened bone quality. Decreased cortical bone mineral density was prominent in HD patients due to the higher levels of PTH compared to those of PD patients^14,17^.

The two most common sites of fracture were the lower limb and the upper limb, regardless of KRT modality. These fracture sites might be related to patients’ predisposition to falls, given their comorbidities and associated complications, such as diabetic neuropathy causing autonomic dysfunction and loss of sensory or motor function^18^.

Another key finding was that most of the initial fractures occurred between 2.5 and 3 years after initiating KRT. Physical activity is generally decreased in dialyzed patients compared with general population^19,20^. Dialysis patients are likely to experience limitation in moderate or vigorous exercise, partly due to prior ESKD comorbidities and mental health issues^21^. Thus, we may educate patients to use extra caution, especially during this period, and emphasize the importance of muscle-strengthening exercise so as to improve balance^22^.

This study also highlights the risk factors for fractures in ESKD patients. Comorbidities, including DM and chronic liver disease, were the same risk factors for fracture reported in the Taiwan’s study^3^. The study suggests that patients with chronic liver disease had a 28% higher risk of fractures, probably due to the lack of ability to convert vitamin D to an 25-hydroxy vitamin D3 form^23^. Two additional identified risk factors in this study were cerebrovascular disease and chronic lung disease (Table 4). We may presume that patients who survived after stroke are at high risk of falls and fractures. Indeed, patients with stroke carried a fourfold increased risk of fracture compared with healthy population^15^. Furthermore, patients with chronic lung disease are more likely to experience fractures, considering the use of inhaled corticosteroids or oral steroids, and their disability to ambulate leading to disuse osteoporosis and fragility fractures^24^.

Osteoporosis is a well-known etiology for fractures in the general population. However, for patients with CKD, another mechanism known as CKD-mineral and bone disorder (CKD-MBD) is associated with an increased risk of fractures^25^. With decreased renal function, increased levels of fibroblast growth factor 23 (FGF-23), hyperphosphatemia, secondary hyperparathyroidism and decreased concentrations of 1,25-hydroxy-vitamin D3 contribute to abnormalities in bone pathology, resulting in fractures ultimately^7^. In addition, exposure to certain medications altering bone metabolism is also known to contribute to bone fractures^7,26^. Thus, we studied the effect of a few commonly prescribed medications on fractures. We identified the use of antidepressants, opioids, gabapentinoids and anti-osteoporotic agents as potential risk factors for fracture, in addition to the use of steroids. Corticosteroid is well known for its deleterious impact on bone mass^27,28^. Patients undergoing treatment with anti-depressants, opioids and gabapentinoids show increased risk of fractures by nearly 19%, 18%, and 21%, respectively (Table 4). Sedation, cognitive impairment and altered sensorium are well-known adverse effects of these medications, thereby increasing the risk of falls and fractures^13,26^. These data are consistent with a previous study by Ishida et al.^29^, suggesting that gabapentinoids were associated with a higher hazard of falls and fractures, and thus should be used with caution, especially in dialyzed patients. Gabapentinoids are known to decrease bone mineral density in the general population, which may predispose to risk of fractures^30,31^. However, the most recent study by Vangala et al.^26^ reported that only the use of opioids was associated with an increased risk of hip fracture (adjusted odds ratio, 1.39; 95% CI, 1.26 to 1.53) in patients exposed to HD, whereas gabapentinoids were not. In addition, the use of drugs for osteoporosis treatment implies severe osteoporosis, suggesting an increased risk of fractures with the anti-osteoporotic drug (HR, 1.17; 95% CIs, 1.02 to 1.33). By contrast, however, the use of vitamin D and its analog, and phosphate binders decreased the risk of fractures by 8% and 4%, respectively (Table 4). The role of vitamin D supplementation in patients with CKD is not clear, but it may enhance bone mineral density by lowering plasma PTH level^32^. The benefit of phosphate binders was not clear from randomized controlled studies until now. However, we speculate that the use of phosphate binders can control secondary hyperparathyroidism in part, and provide calcium supplements using calcium-containing phosphate binders^5,33^. Therefore, the possible protective role of vitamin D and phosphate binders deserves further investigation.

This study has several limitations. First, since the diagnosis of fractures, co-morbidities, and ESKD in our study was solely dependent on the diagnostic codes, and specific codes (V001, V003, V005) from claims data, those who inadvertently missed these codes could be excluded from the study. In addition, by excluding patients with history of previous fracture, which may indicate the decreased bone mineral density, it may influence our main results inversely, that is, by underestimating fracture risks in ESKD patient. Second, we were unable to determine the factors associated with fractures, such as biochemical parameters reflecting nutritional status, body mass index, muscle mass, patient compliance, or menopause, due to the nature of the HIRA database. Also, information about individual’s activity level and the ability to ambulate, which were related to the risk of falls, was not available from our database. Lastly, as this study was a single nation study, the results may not be generalized to other populations. However, this study was based on the NHIS database enrolling a large patient population, suggesting reliability of the data collected.

In conclusion, this study showed that patients exposed to HD carry the highest risk, followed by those undergoing PD and KT, and the two most common fracture sites were the lower limb and upper limb. The average time of the first fracture after the initiation of KRT was between 2.5 and 3 years, which can be utilized in fracture prevention education. Diabetes mellitus, cerebrovascular disease, chronic lung and liver disease were risk factors of fractures. The use of steroids, anti-osteoporosis medications, and some classes of psychotropics and opioids was associated with an elevated risk.

## Supplementary Information

Supplementary Information.

## Data Availability

Data available on request duet to the policy of HIRA. The data presented in this study are available on request from the corresponding author. The data are not publicly available due to the policy of HIRA.
