# Supplementary material for: Characteristics of fracture in patients who firstly starts kidney replacement therapy in Korea: a retrospective population-based study
Source: Sci Rep. 2022 Feb 24;12:3107. doi: 10.1038/s41598-022-07178-4 (PMC8873500; doi:10.1038/s41598-022-07178-4)

**Supplementary materials**

A. (a) multicollinearity between covariates and (b) validity of model fitting for cox proportional regression

**Variance inflation factors (VIF)**

|  | **VIF** |
| --- | --- |
| Age at index, years | 1.24 |
| Sex, female | 1.02 |
| KRT modality |  |
| HD | 2.35 |
| PD | 2.11 |
| KT |  |
| Comorbidities |  |
| Diabetes | 1.19 |
| Hypertension | 1.14 |
| Cardiovascular | 1.10 |
| Cerebrovascular | 1.07 |
| Chronic lung disease | 1.06 |
| Chronic liver disease | 1.01 |
| Medication at baseline |  |
| Steroid | 1.07 |
| Vitamin D and its analog | 1.07 |
| Phosphate binders | 1.11 |
| Anti-osteoporotic medications | 1.01 |
| Anti-depressants | 1.05 |
| Opioids | 1.04 |
| Gabapentinoids | 1.07 |

**Testing Global Null Hypothesis:** $\boldsymbol{\beta=0}$

| Test | Chi-Square | Degree of Freedom | *P* > ChiSq |
| --- | --- | --- | --- |
| Likelihood Ratio | 2049.319 | 17 | <.0001 |
| Score | 2003.384 | 17 | <.0001 |
| Wald | 1959.404 | 17 | <.0001 |

B. Scaled Schoenfeld residuals plot by time


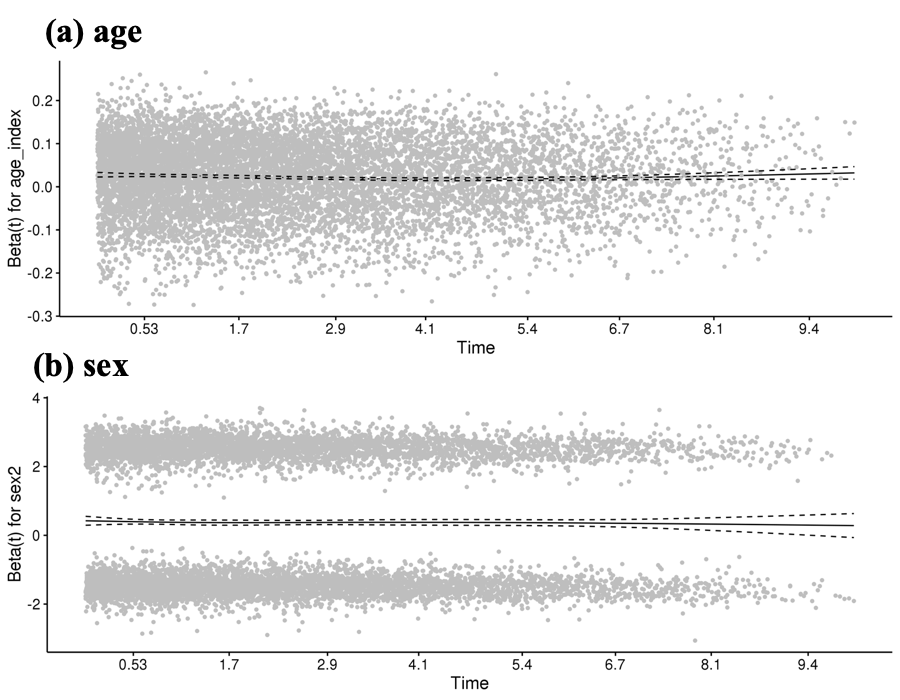


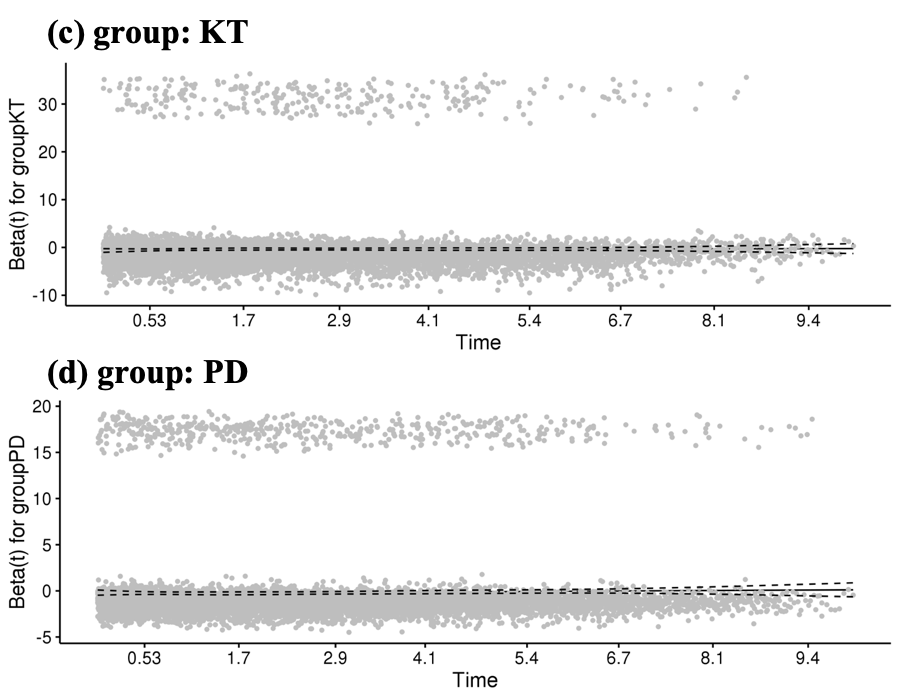


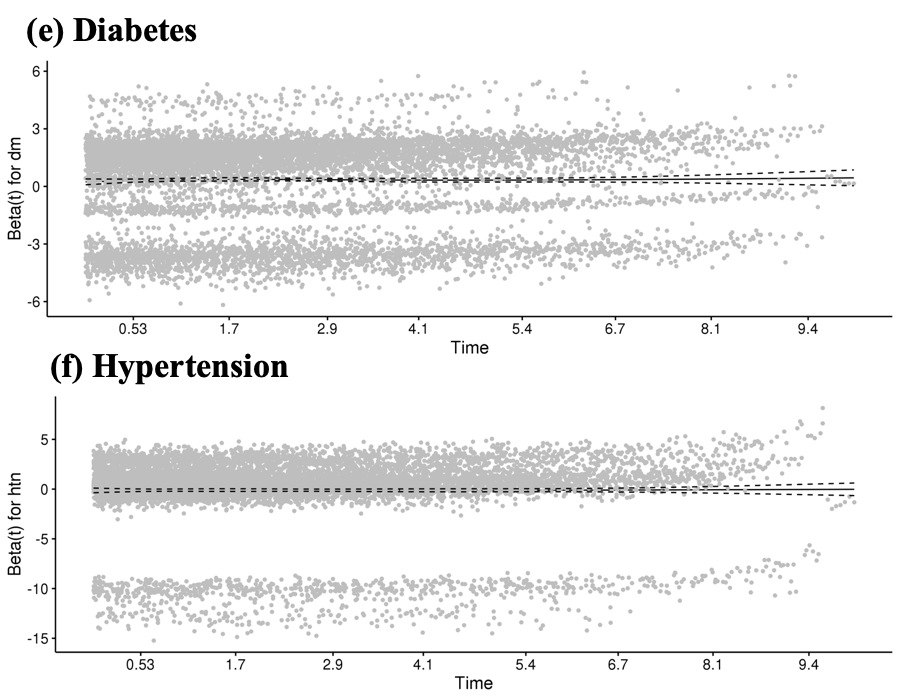


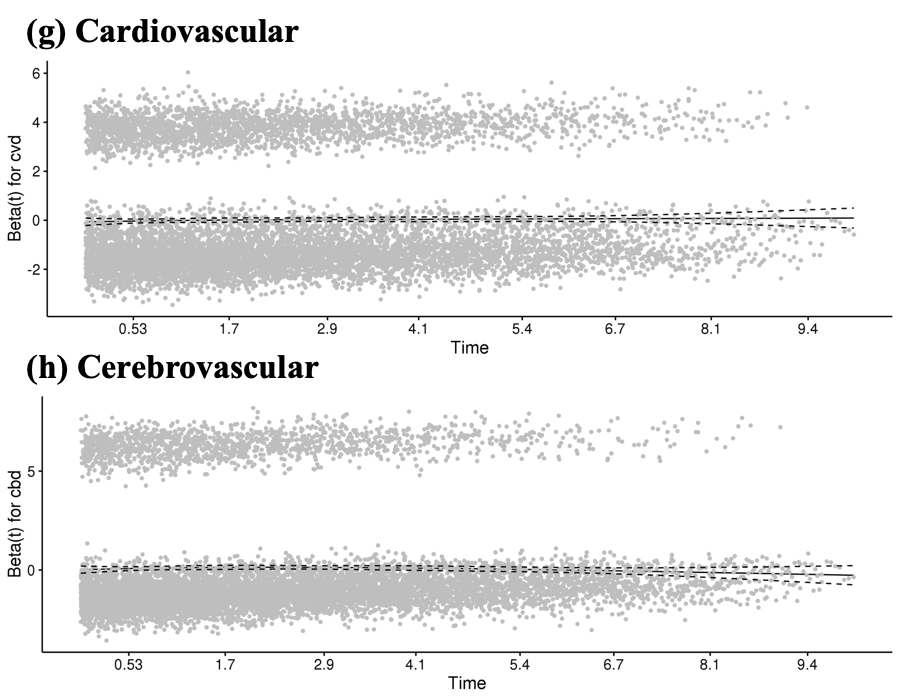


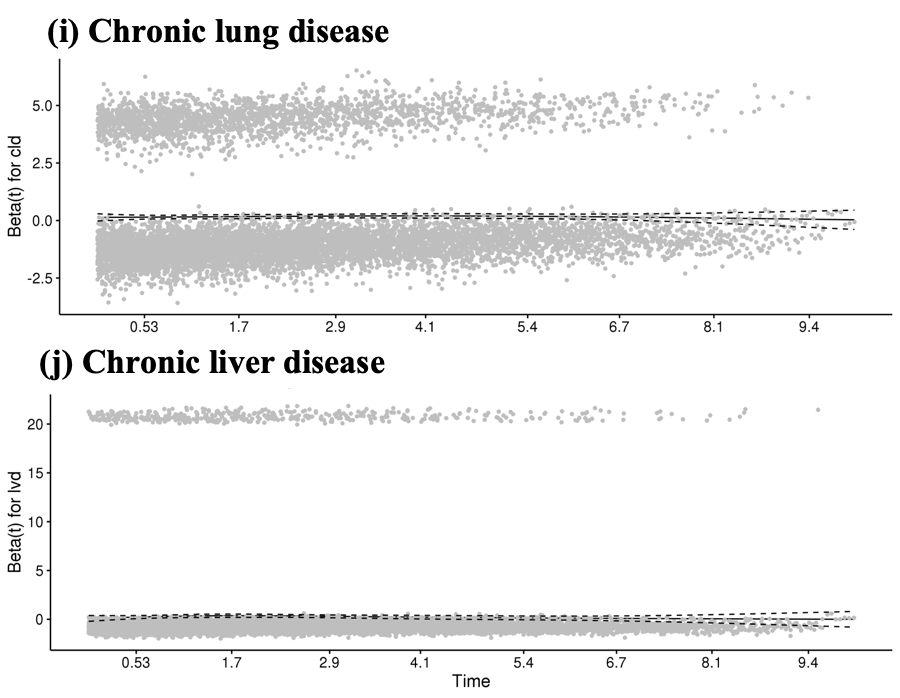


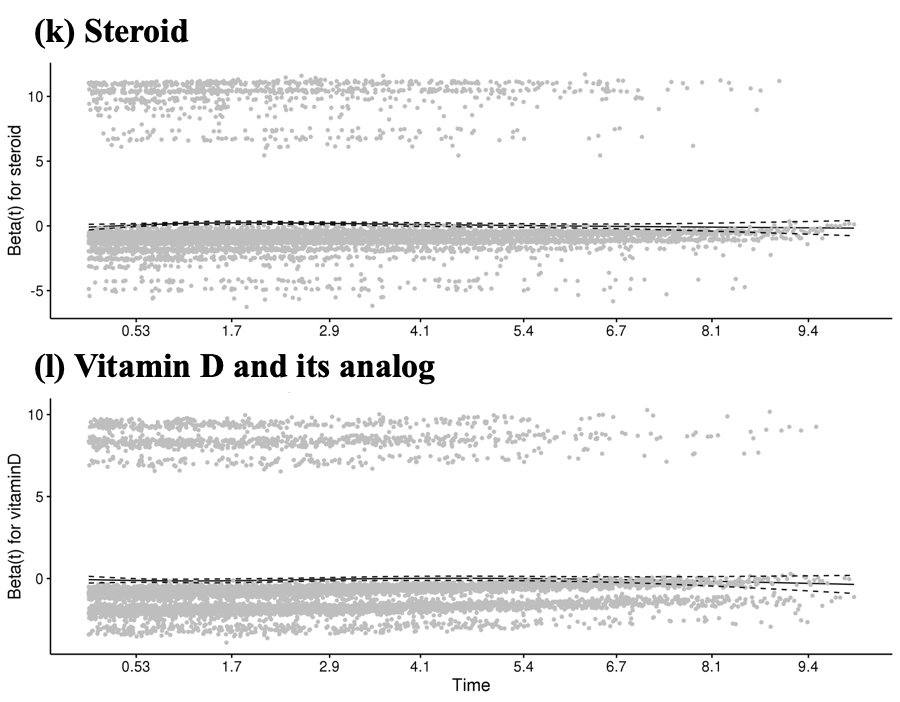


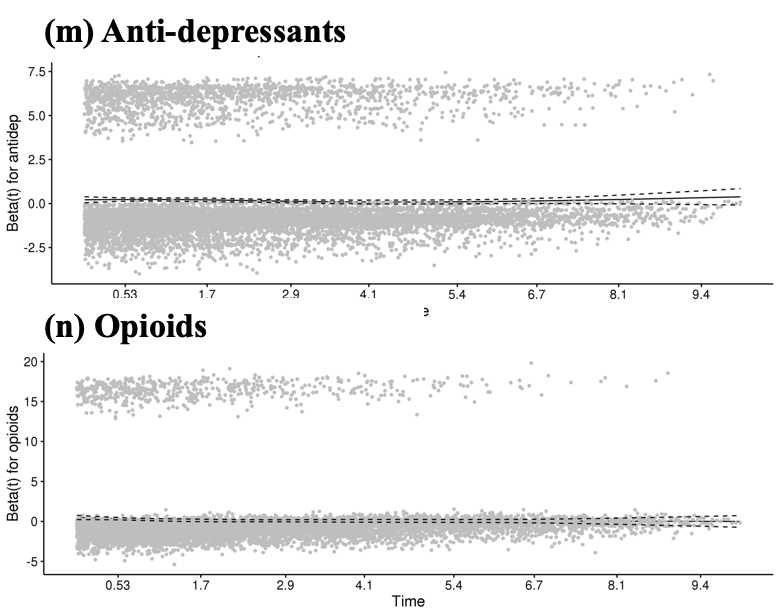


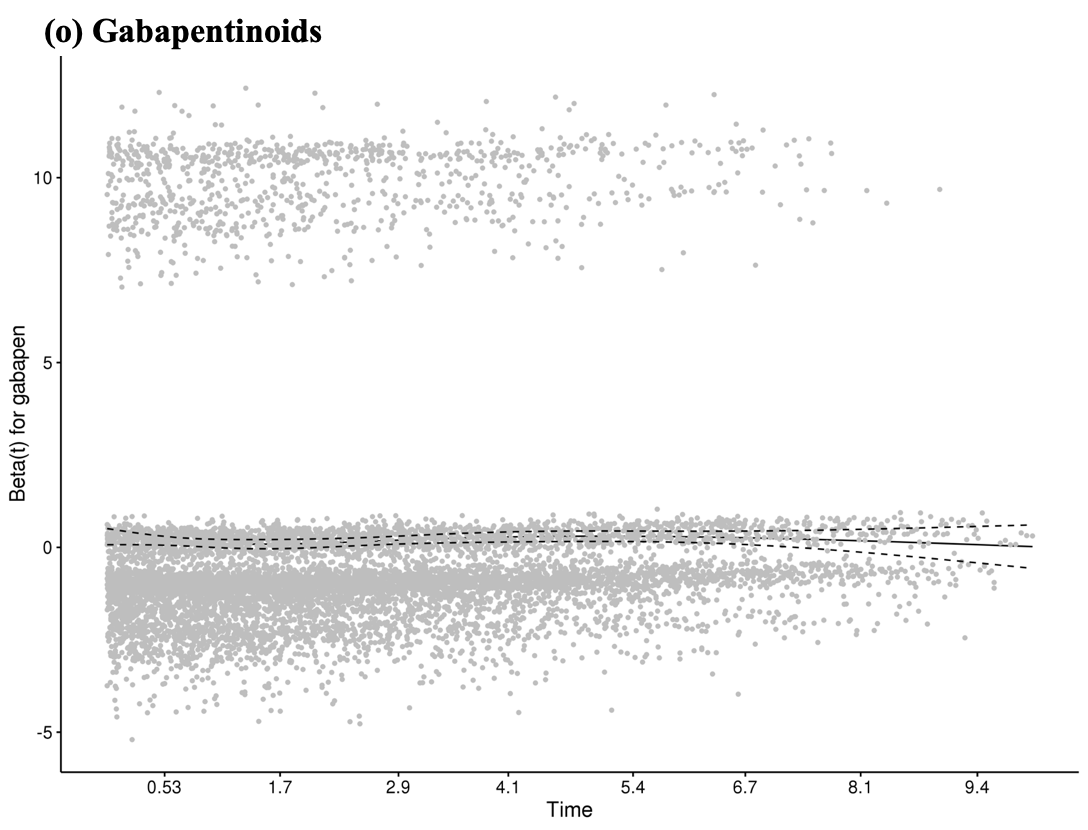

Supplement: Supplementary file 1 — Supplementary Information. [file 41598_2022_7178_MOESM1_ESM.docx]
